# Examining the Metropolitan and Non-metropolitan Educational Divide: Science Teaching Efficacy Beliefs and Teaching Practices of Australian Primary Science Educators

**DOI:** 10.1007/s11165-023-10113-w

**Published:** 2023-05-02

**Authors:** James Deehan, Amy MacDonald

**Affiliations:** Faculty of Arts and Education, School of Education, Charles Sturt University, Panorama Avenue, Bathurst, 2795 Australia

**Keywords:** Science education, Primary education, Regional, Remote, Efficacy beliefs, Teaching Practices

## Abstract

The provision of quality science education is a global priority beset by longstanding challenges, which can be amplified in rural and regional contexts. This creates a dual problem where stakeholders must focus on the improvement of science education outcomes whilst being cognisant of the established divided between metropolitan and non-metropolitan learners. Considering the recent positive TIMSS results which showed equitable science results for regional, remote and metropolitan Australian year 4 students, this paper aims to examine the relationship between primary teachers’ school location and their science teaching efficacy beliefs and reported science teaching practices. A total of 206 Australian primary science educators responded to a cross sectional quantitative survey. Descriptive statistics, analysis of variance (ANOVA) and chi-square analyses found no statistically significant differences between metropolitan and non-metropolitan teachers on measures of science teaching efficacy beliefs and reported science teaching approaches. This apparent contradiction of established research themes merits deeper school and student-focused research to understand the practical implications that could arise from these findings.

## Introduction and Literature Review 

Many primary aged learners are limited in their capacity to apply the knowledge and skills gained in their formal science education experiences in ways that make meaning beyond the classroom. Evidence from the Trends in International Mathematics and Science Study (TIMSS) provides robust, if fallible, evidence that most year 4 students across the majority of participating nations have difficulty in generalising their science learning to new contexts (Martin et al., 1997; Thomson et al., 2020a). The most recent iteration of the Australian Sample Assessment in Science Literacy also indicated limitations among primary students’ science knowledge and capabilities (ACARA, 2019) that can persist after the primary years (Thomson & De Bortoli, 2008; Thomson et al., 2019). It can reasonably be argued that systems of science education could be improved in terms of developing learners’ scientific literacy: a central tenant of science education research (Roberts & Bybee, 2014; NASEM, 2016) that is embodied in science curricula globally (ACARA, 2021a; Eggleston, 2018; Kim et al., 2013; NGSS, 2013). This study adopts a broad definition of scientific literacy as a learners’ ability to apply science knowledge and skills to novel contexts whilst recognising the wide-ranging socio-cultural impacts of science advancements (Bybee, 1997; Roberts & Bybee, 2014).

Primary teachers have the most direct role in developing children’s scientific literacy, but they themselves can often face hurdles from their experiences as science learners (Harms & Yager, 1981; Howitt, 2007). Indeed, despite their willingness to pursue engaging, student-centred science teaching practices (ACARA, 2019; 2013; Banilower, 2019), primary teachers can still be limited by their science content knowledge (Appleton, 2003; Murphy & Smith, 2012) and low science teaching efficacy beliefs (STEBs) (Denessen et al., 2015). These issues have been associated with an overreliance on passive, disengaging practices such as note taking, lectures and teacher-driven investigations (Goodrum et al., 2001; Goodrum & Rennie, 2007; Tytler et al., 2008), in addition to insufficient classroom time to satisfy minimum science curricular requirements (Goodrum et al., 2001; Office of the Chief Scientist, 2012; Tytler & Griffiths, 2003; Tytler et al., 2008). Extant literature suggests that these issues are global in scope (Carlone et al., 2011; Osborne & Dillon, 2008; Roth, 2014; Weiss et al., 2003).

These challenges can be compounded by inequitable educational experiences and outcomes between metropolitan and non-metropolitan students (Cardak et al., 2017; Cooper et al., 2018; Cuervo & Acquaro, 2018; Halsey, 2018; OECD, 2013). For at least half a century, research has consistently shown that rural, regional and remote learners (hereafter referred to as non-metropolitan learners) experience greater disadvantage than learners enrolled in metropolitan schools (Human Rights & Equal Opportunity Commission, 2000). Non-metropolitan education can often be diminished by poorer teacher retention, more inexperienced and ‘out-of-field’ teachers, and less relevant curricula, which often results in lower levels of educational attainment and lower likelihood of pursuing higher education (Cardak et al., 2017; Cooper et al., 2018).

Science achievement is no different, with PISA and TIMSS data showing significant differences in the performance of metropolitan and non-metropolitan learners (Fraser et al., 2019; Sullivan et al., 2018). Science education gaps are likely related to staff capacity, resource availability and challenges to learning environments in non-metropolitan schools (Cuervo & Acquaro, 2018; Halsey, 2018; Sullivan et al., 2018). In accordance with trends in the field, the year 4 mathematics, year 8 mathematics and year 8 science assessments in the most recent iteration of the TIMSS all yielded substantial gaps between metropolitan and non-metropolitan schools (Thomson et al., 2020b). For example, the percentages of year 8 science students from regional (67%) and remote (50%) locations at or above the National Proficiency standard were below those from metropolitan centres (77%). Curiously, for the year 4 science assessment, the National Proficiency percentages of remote (74%), regional (75%) and metropolitan (80%) were similar as no statistically significant difference between metropolitan and non-metropolitan was detected (Thomson et al., 2020b). When considered in conjunction with the significant overall increase in Australian year 4 students’ science achievement (Thomson et al., 2020a), further research into these trends of improved educational attainment and geographical equity is warranted. It may be the case that long-term efforts, by a variety of stakeholders, are beginning to influence the quality of Australian primary science teaching (e.g. Deehan, 2021, 2022; Fraser et al., 2019; Fitzgerald et al., 2021; Skamp & Preston, 2021).

It is possible that the recent evidence of more equitable primary science performance between metropolitan and non-metropolitan learners (Thomson et al., 2020a, 2020b) could be reflective of the dissemination of more evidence based (Aubusson et al., 2015, 2019; Deehan et al., 2022), conceptually clear science teaching practices (Harlen, 2015; Roth, 2014) through Initial Teacher Education (ITE) programmes (Deehan, 2021, 2022; Fitzgerald et al., 2021). Such a claim cannot be investigated without an explicit understanding of what may constitute ‘best practice’ in primary science education. Thus, Appendix 1 presents a list of 38 evidence based primary science teaching approaches (Aubusson et al., 2015, 2019; Deehan, 2017, 2022) which serves as a foundation for the investigation of primary science education across metropolitan and non-metropolitan contexts in this paper. The following question will be answered in the paper:

- Are there differences in the reported science teaching practices and science teaching efficacy beliefs of a sample Australian primary teachers based on school location (metropolitan and non-metropolitan)?

## Theoretical Framework

The concept of teacher efficacy has been utilised as a major theoretical underpinning for this study due to its close association with classroom practices and outcomes, which cannot be directly investigated through distal data, and its well-established literature base spanning science education research (Enochs & Riggs, 1990; Riggs & Enochs, 1990), education research (Gibson & Dembo, 1984; Goddard et al., 2000) and psychology research (Bandura, 1977, 1986). Bandura’s seminal unifying theory of behavioural change emphasised the importance of self-efficacy in influencing coping behaviours, exertion of effort and resilience in the performance of desirable actions (Bandura, 1977). Self-efficacy is not an immutable characteristic as it has been shown to vary based on positive or negative influences, with Bandura (1997a, 1997b) himself citing mastery experiences (ME), vicarious experiences, verbal persuasion and emotional arousal (EA) as being the strongest influences on self-efficacy. It is clear such factors would be having complex impacts on teachers’ self-efficacy in all educational settings. Bandura also transitioned the concept of efficacy to teaching practice where he found that ME had the strong impact on teachers’ self-efficacy (Bandura, 1997a, 1997b). This connection between self-efficacy and firsthand MEs provides a reasonable theoretical justification for examining teacher efficacy and reported teaching practices. At the very least, Bandura’s extensive body of work (e.g. Bandura, 1977, 1986, 1997a, 1997b) shows that self-efficacy is a construct that is both associated with desirable actions and malleable, which enhances its conceptual value in educational research. Teacher efficacy (TE) is a measure of an educator’s beliefs in their own and/or their profession’s ability to enhance student learning outcomes (Gibson & Dembo, 1984). TE is a valuable proxy measure in education because higher TE can be indicative of more committed teachers (Chesnut & Burley, 2015; Høigaard et al., 2012) who adopt more effective teaching practice (Klassen & Tze, 2014; Nie et al., 2013) that often result in strong outcomes for their learners (Çoğaltay, & Karadağ, 2017; Goddard et al., 2000). TE has also been consistently operationalised through valid and reliable measures (e.g. Bandura, 1997a, 1997b; Humphries et al., 2012; Lumpe et al., 2000).

The Science Teaching Efficacy Belief Instruments (STEBI-A and STEBI-B) have been consistently employed as measures of inservice and preservice teachers’ STEBs for over three decades (Enochs & Riggs, 1990; Riggs & Enochs, 1990) through a wide variation of research contexts (Deehan, 2017). Like the TE research, the STEBI research has linked primary teachers’ STEBs to desirable outcomes for both teachers and students (Deehan et al, 2020; Clark, 2009). In particular, teachers with higher STEBs are more likely to use more active teaching (Burton, 1996; Lardy, 2011), feel more positively towards their school leaders (Clark, 2009) and, most importantly, improve the science learning outcomes of their students (Angle and Moseley, 2009). There is also a clear gap in addressing the metropolitan and non-metropolitan divides in the STEBI literature as none of the 257 articles considered in a recent meta-analysis addressed this important area of educational equity (Deehan, 2017).

## Methodology

A digital survey was used to investigate the potential teaching and efficacy differences between metropolitan and non-metropolitan educators in a jurisdiction of close to 30,000 inservice primary teachers employed across slightly more than 1500 public schools. Quantitative data were collected from an online survey of educators’ primary science teaching practices and efficacy beliefs from mid-to-late 2021.

### Context

There were 173 schools (> 10%) from the target population represented in this project. The Australian Curriculum and Reporting Authority (ACARA, 2021b) designates each Australian school’s geolocation based on the five levels of the Australian Statistic Geography Standard (ASGS) Remoteness Structure (ABS, 2016): (1) Major Cities, (2) Inner Regional, (3) Outer Regional, (4) Remote and (5) Very Remote. Table 1 shows the geolocation distribution of the schools sampled versus the target population. The geographical locations are reasonably similar, although the sample is more skewed towards regional locations than the target population. There was no statistically significant difference in the mean Index of Community Socio-Educational Advantage (ACARA, 2016) scores of the sampled and non-sampled schools, *t*(1594) = 1.845, *p* = 0.418, meaning that the sampled schools did not vary considerably from the target population in terms of parental occupation, parental education, school remoteness and Indigenous student enrolment percentages.

**Table 1:** Geolocation distributions of the sample schools (*n* = 173) and the target population (*n* > 1500)

	Sample schools %	Target population %		Sample schools %	Target population %
Major Cities	47.5%	53.0%	Metropolitan	47.5%	53.0%
Inner Regional	30.5%	28.5%	Non-metropolitan	52.5%	47.0%
Outer Regional	19.5%	16.5%			
Remote	2.0%	1.5%			
Very Remote	0.5%	0.5%			

### Participants

A series of recruitment approaches were employed as part of a non-probabilistic, purposive sampling of the target population of primary teachers. Two email invitations were sent to each school in the final two semesters of the 2021 school year. Physical mailouts, with QR codes to access the online survey, were sent to each school between email invitations. These primary recruitment strategies were supplemented by opportunistic snowball sampling and sharing across online platforms, both professional and social.

The final sample of 206 primary teachers, representing 0.67% of the target population, was strong in statistical terms (VanVoorhis & Morgan, 2007), and the sampling ratio compares favourably to seminal work in this space (1:150) (Goodrum et al., 2001). Whilst sampling remains ungeneralisable, the characteristics of the sample afford some broader speculative interpretations of the findings. Table 2 summarises the demographic data for all 206 participants.

**Table 2:** Participant demographic data (*N* = 206)

Gender							
Female				**Male**			
153				43			
Age							
18–24	25–34		**35–44**	**45–54**		**55–64**	**65 + **
3	60		53	57		28	5
Role							
Classroom teacher		**Administrator**			**Both**		
173		13			20		
Employment status							
Full time		**Fixed term contract**			**Casual**		
133		66			7		
School location							
Metropolitan		**Non-metropolitan**			**Unspecified**		
91		109			6*		
Averages							
Years of experience		**School ICSEA**			**School ICSEA %**		
15.4		983.8			41.6		

*The unspecified participants did not identify specific schools but provided email addresses which indicated their status as part of the target population

### Quantitative Survey

The online survey was comprised of three key areas: the STEBI-A, science teaching approaches and curriculum coverage.

#### The STEBI-A

A selection of 16 5-point Likert scale items comprised the Personal Science Teaching Efficacy (PSTE) beliefs and Science Teaching Outcome Expectancies (STOE) scales (Riggs & Enochs, 1990). The eight PSTE items (e.g. ‘I generally teach science effectively’) are added together to measure participants’ beliefs about their personal effectiveness in science teaching. The eight STOE items (e.g. ‘The inadequacy of a student’s science background can be overcome by good teaching’) measure participants’ more general beliefs about science teaching to affect student learning positively. Upon initial publication, both the PSTE (α = 0.92) and STOE (α = 0.74) scales were valid, with the discrepancy being replicated (e.g. Moslemi & Mousavi, 2019) and discussed in the STEBI literature (Deehan, 2017; Unfried et al., 2022). The PSTE (α = 0.89) and STOE (α = 0.74) scales were reliable in this project (Pallant, 2020).

#### Science Teaching Approaches

The 38 science teaching approaches from Appendix 1 were presented to participants to dichotomously identify which they utilised in their science teaching practice. They were also afforded the opportunity to identify any additional approaches from their science teaching repertoires that they felt were not represented in the framework. The research teams coded the open responses to remove inappropriate (e.g. ‘Just do it’) and redundant (e.g. ‘Project-based learning’) responses. Open responses deemed appropriate by the research team included resource suites (e.g. ‘Inquisitive’), science fairs and integrated STEM approaches. Each participant was assigned a metric ‘Total Approaches’ score based on the number of relevant approaches selected or otherwise identified in their response.

#### Curriculum Coverage

Respondents were asked to identify all of the areas of the science curriculum they had addressed in their teaching during the past year. Eleven dichotomous items covered the strands and sub strands of the current Australian K-10 Science Curriculum (ACARA, 2021a). Table 3 organises the 11 curriculum areas presented to participants under the Science Understanding, Science as a Human Endeavour and Science Inquiry Skills. The maximum score on the Curriculum Coverage measure was 11.

**Table 3:** Australian K-10 science strands and sub strands

Science understanding	Science as a human endeavour	Science inquiry skills
Biological Sciences Chemical Sciences Earth and Space Sciences Physical Sciences	Nature and Development of Science Use and Influence of Science	Questioning and Predicting Planning and Conducting Processing and Analysing Data and Information Evaluating Communicating

### Data Analyses

Initially, descriptive statistics were calculated for the measures of science teacher efficacy (i.e. PSTE and STOE) and reported science teaching approaches (i.e. total approaches and curriculum coverage) for the groups of metropolitan or non-metropolitan-based teachers. To determine the difference between the two groups, a one-way ANOVA was computed on the PSTE, STOE, Total Approaches and Curriculum Coverage variables (Pallant, 2020). For additional detail, the magnitude of differences was measured through Hedge’s G to account for the different group sizes. The context, data and variance assumptions for the ANOVA were not violated, at least in part due to the resilience afforded by the large total sample size (VanVoorhis & Morgan, 2007). Non-parametric chi-squares were conducted for the sake of a more thorough interrogation of any between group differences on the reported use of the 38 separate science teaching approaches (Appendix 1).

## Results—Are There Differences in the Reported Science Teaching Practices and Science Teaching Efficacy Beliefs of a Sample Australian Primary Teachers Based on School Location (Metropolitan and Non-metropolitan)?

There are very few observable differences between the metropolitan and non-metropolitan groups on the PSTE, STOE, Total Approaches and Curriculum Coverage measures. Table 4 presents the descriptive statistics for the non-metropolitan and metropolitan teachers’ PSTE, STOE, Total Approaches and Curriculum Coverage scores. On both efficacy scales, the metropolitan and non-metropolitan educators displayed similar means and standard deviations, with both groups falling clearly into the ‘somewhat efficacious’ category (i.e. > 24 and < 32). In accordance with much of the existing STEBI literature (Deehan, 2017), the PSTE scores were higher than the STOE scores for both groups. The PSTE scale was the only measure with a between group mean difference greater than one point (1.06). The mean score differences between the metropolitan and non-metropolitan groups on the STOE subscale (0.18) and the Total Approaches measure (0.25) appeared negligible. In fact, Curriculum Coverage did not differ at all between the two geolocation groups.

**Table 4:** Descriptive statistics for the STEBs of metropolitan- and non-metropolitan-based teachers

Variable	Group	*N*	Mean	Std. deviation
PSTE	Non-metropolitan	109	30.05	6.068
	Metropolitan	91	31.11	4.639
STOE	Non-metropolitan	109	29.48	3.686
	Metropolitan	91	29.30	3.345
Total Approaches	Non-metropolitan	109	17.57	6.425
	Metropolitan	91	17.32	6.880
Curriculum Coverage	Non-metropolitan	109	8.65	2.070
	Metropolitan	91	8.65	2.013

There were no statistically significant differences detected between the metropolitan and non-metropolitan groups on the PSTE, STOE, Total Approaches and Curriculum Coverage measures. Table 5 presents the output for a one-way ANOVA on the four dependent variables between the geolocation groups. Despite the effect size (*g* = 0.194) indicating a small advantage to the metropolitan teachers on the PSTE scale, the difference was not significant (*p* = 0.162). Additionally, there were no statistically significant between-group differences on the STOE subscale (*p* = 0.720), the Total Approaches reported (*p* = 0.791) and the Curriculum Coverage scores (*p* = 0.992).

**Table 5:** One-way ANOVA for STEBs, Total Approaches and Curriculum Coverage by geographical location (metropolitan versus non-metropolitan)

Variable	Source	SS	df	MS	*F*	*p*	ES (Hedges’ *g*)
PSTE	Between groups	56.148	1	56.148	1.880	0.162^ ns^	− 0.1939
	Within groups	5913.672	198	29.867			
STOE	Between groups	1.613	1	1.613	0.129	0.720^ ns^	0.0509
	Within groups	2474.182	198	12.496			
Total Approaches	Between groups	3.103	1	3.103	0.070	0.791^ ns^	0.0378
	Within groups	8718.492	198	44.033			
Curriculum Coverage	Between groups	0.000454	1	0.000454	0.000109	0.992^ ns^	0.0010
	Within groups	827.499546	198	4.179291			

*ns* not significant, *SS* sum of squares, *MS* mean of squares

More precise chi-square analyses were conducted on participants’ responses to the 38 specific framework approaches, and the number of ‘other’ approaches identified to account for the lack of sensitivity inherent in the broad Total Approaches measure. Table 6 summarises the statistical output for the series of chi-square tests computed to ascertain the differences between the metropolitan and non-metropolitan teachers on the reported use of specific science teaching approaches. In an extension of the results of the one-way ANOVA presented above, there were no significant differences between the groups in the reported uptake of 95% of the specific approaches, including the rate of identification of ‘other’ approaches, a possible indication of similar approaches to science teaching in metropolitan and non-metropolitan primary schools. There were only two teaching approaches that differed in frequency between the two groups. The non-metropolitan educators were more likely to include peer tutoring in their science teaching repertoires, *X*^2^ (1, *N* = 200) = 5.518, *p* = 0.019, whereas their metropolitan counterparts reported using Debate strategies with comparatively greater frequency, *X*^2^ (1, *N* = 200) = 4.765, *p* = 0.029. However, the significance of these findings in practice is contestable due to the lower overall frequency of use for Peer Tutoring and Debate approaches.

**Table 6:** Chi-square analyses of teaching approaches by the metropolitan (*n* = 91) and non-metropolitan (109) groups (counts and percentages)

Approach	NMet count (%)	Met count (%)	*X*^2^	Sig	Approach	NMet count (%)	Met count (%)	*X*^2^	Sig
Hands on tasks	103 (95%)	87 (96%)	0.128	0.720	Excursions	42 (39%)	40 (44%)	0.603	0.437
Group work/cooperative learning	103 (95%)	85 (93%)	0.104	0.747	Predict-Observe-Explain (POE) cues	37 (34%)	36 (40%)	0.675	0.411
Class discussions	101 (93%)	80 (88%)	1.301	0.254	Diagnostic assessment for alternative conceptions	33 (30%)	28 (31%)	0.006	0.940
Teacher demonstration	97 (89%)	83 (91%)	0.271	0.603	Station Rotation	28 (26%)	28 (31%)	0.635	0.425
Watching videos	101 (93%)	79 (87%)	1.884	0.170	The 5Es Framework	31 (28%)	24 (26%)	0.106	0.744
Big ideas/inquiry questions	92 (84%)	71 (78%)	1.340	0.247	Other	26 (24%)	18 (20%)	0.391	0.080
Inquiry learning	85 (78%)	71 (78%)	0.000	0.995	Science in the Media	23 (21%)	27 (30%)	1.942	0.163
Joint construction	78 (72%)	66 (73%)	0.023	0.879	Individual Reading	24 (22%)	19 (21%)	0.038	0.845
Student-centred investigations	78 (72%)	65 (71%)	0.000	0.984	Deep Reflection	21 (19%)	14 (15%)	0.518	0.472
Modelling	76 (70%)	60 (66%)	0.328	0.567	Guest Speakers	24 (22%)	11 (12%)	3.388	0.066
Outdoor science	68 (62%)	61 (67%)	0.468	0.494	Community Projects	19 (17%)	14 (15%)	0.151	0.698
Open/higher order questioning	66 (61%)	59 (65%)	0.388	0.533	Peer Tutoring	21 (19%)	7 (8%)	5.518	0.019*
Worksheets	68 (62%)	56 (62%)	0.015	0.902	Debate	9 (8%)	17 (19%)	4.765	0.029*
Project/problem-based learning	70 (64%)	47 (52%)	3.229	0.072	Nature of Science Teaching	12 (11%)	14 (15%)	0.639	0.360
Teacher lead investigations	62 (57%)	50 (55%)	0.075	0.784	Constructivism	11 (10%)	11 (12%)	0.202	0.653
Open/guided discovery	62 (57%)	45 (49%)	1.101	0.294	Claim-Evidence-Reasoning (CER) Cues	5 (5%)	9 (10%)	2.142	0.143
Digital technology/simulations	59 (54%)	46 (51%)	0.255	0.614	Second Hand Research	6 (6%)	7 (8%)	0.391	0.532
Direct instruction/transmission	59 (54%)	45 (49%)	0.435	0.510	Analogies (Content Representations)	5 (5%)	7 (8%)	0.848	0.357
Cross curricular integration	54 (50%)	37 (41%)	1.578	0.209	Lectures	2 (2%)	6 (7%)	2.925	0.087
Note taking	52 (48%)	40 (44%)	0.281	0.596					

df = 1 for all analyses; * denotes results significant at the 0.05 level; ** denotes results highly significant at the 0.01 level

## Discussion

The findings presented in this paper seem to align with the most recent Australian TIMSS results, which showed no statistically significant differences in metropolitan, regional and rural year 4 students’ science achievement (Thomson et al., 2020b), as there were no substantial differences between metropolitan and non-metropolitan primary teachers’ PSTE, STOE, Total Approach and Curriculum Coverage scores. Even further interrogation of between-group differences for specific teaching practices revealed that reported use rates were similar for 95% of the approaches presented in the framework. A tentative interpretation may be that the similar STEBs and reported science teaching practices of metropolitan and non-metropolitan primary teachers may be influencing more equitable year 4 science outcomes by school location according to the TIMSS (Thomson et al., 2020b). Perhaps, such findings reflect the efforts of teachers, researchers and other educational stakeholders to improve the quality and equity of primary science education (e.g. Aubusson et al., 2015; Aubusson et al., 2019; Fitzgerald et al., 2021; Skamp & Preston, 2021). Indeed, interviews and surveys from a group of 17 primary science academics, supplemented by analysis of publicly available documents, indicated that authentic, accessible and student-centred practices are central in Australian pre-service primary science education (Deehan, 2022). However, any interpretation should be made with a high degree of caution as there is no classroom or student data to confirm reported practice or elucidate how teaching practices relate to student outcomes. Cautious optimism is the best way to interpret these findings as they contradict much of the educational research signalling rural and regional disadvantage. As outlined in the introduction, metropolitan learners have long experienced better short- and long-term educational outcomes than their peers in the regional and rural areas. Recent trends towards equity in Australian primary science warrant further investigation. At the very least, it appears that teachers may be resilient to issues surrounding place, and thus have tremendous potential to contribute to the long-term closing of metropolitan and non-metropolitan educational divides.

There are a number of viable research pathways that could build on this study. First, the absence of significant differences in primary teachers’ STEBs and reported practices between metropolitan and non-metropolitan locations, particularly when considered alongside the equitable rural, regional and metropolitan year 4 science achievement levels in the most recent TIMSS (Thomson et al., 2020b), merits deeper investigation to determine if these findings are aberrations or could inform discourse and decisions surrounding long-standing gaps in metropolitan and non-metropolitan educational outcomes. In particular, the experiences and perspectives of primary science educators and students alike could help to further clarify the nature of geographical location as it relates to primary science education. Second, the data presented in this project represents a single public education jurisdiction in Australia. This means that research at a national scale is needed to determine if these findings are part of a larger pattern of bridging rural, regional or metropolitan divides, or whether the findings are an aberration related to other educational factors, such as teacher traits, funding, resources and others. Similar research should also be pursued to position these findings within a global context. Third, school and classroom level data are vital to addressing the issue of ecological validity commonly associated with large scale quantitative research projects in education (Gorur, 2017) by providing more nuanced, detailed information.

Any interpretation of the findings presented in this manuscript should be tempered by a full understanding of the methodological limitations. Despite providing some useful and methodological defensible insights, the quantitative operationalisation of school locations unavoidably fails to capture the complexity of the lived experiences of those who live and learn in both metropolitan and non-metropolitan communities. Although the ASGS remoteness structure (ABS, 2016) is widely adopted, it cannot cater for issues such as for individual movement between jurisdictions and requires categorisation that may not have any tangible meaning in practice, thus creating an artificial sense of accuracy. Indeed, many educators are likely to have completed their ITE in metropolitan areas and thus may have rural and regional experiences and perspectives that differ from those with more long-term connections to their communities. The importance of the metropolitan- and non-metropolitan-focused analyses presented in this paper should not be overstated as place inherently intersects with factors such as socio-economic status and gender, in ways that were not considered. Additionally, the absence of students as a data source prevents a clear link between the educators’ STEBs and teaching practices, and student outcomes from being established without relying heavily on the theoretical framework (Efficacy). Also, despite the rigour of the framework of approaches (Appendix 1) underpinning this paper, it can neither be a complete reflection of all the approaches that may comprise a primary science teachers’ professional and pedagogical experience repertoire (Loughran et al., 2001, 2004) nor can it capture the complex ways that approaches are instigated and altered in classroom settings. Finally, despite the relative strength of the participant sample, the non-probabilistic recruitment techniques prevent full generalisation of the findings.

## Conclusion

The findings presented in this paper contradict much of the existing literature that has described educational divides between metropolitan and non-metropolitan learners. When considered alongside the outlying equitable TIMSS science achievement for Australian year 4 students across regional, rural and metropolitan centres (Thomson et al., 2020a, 2020b), the similar STEBs and reported science teaching between metropolitan and non-metropolitan educators could be indicative of an emerging trend of geographic equity in Australian primary science education. It is important for students to be supported by efficacious teachers who, regardless of school location and status, can overcome general and localised challenges to the provision of high-quality science education (Bandura, 1977, 1986, 1997a, 1997b). This research has shown that both metropolitan and non-metropolitan learners may be equally likely to experience the benefits associated with higher teacher efficacy, including stronger teaching practice (Burton, 1996; Klassen & Tze, 2014; Lardy, 2011; Nie et al., 2013) and better student-outcomes (Angle and Moseley, 2009; Çoğaltay, & Karadağ, 2017; Goddard et al., 2000). The STEB findings presented in this paper are particularly important as non-metropolitan teachers have historically faced considerable challenges in the provision of high quality education (Cardak et al., 2017; Cooper et al., 2018; Cuervo & Acquaro, 2018; Halsey, 2018; OECD, 2013). Whilst the non-probabilistic sampling and reliance on distal data prevent any definitive statements from being made at this time, there is a clear need for further research in this space as there may be insights relevant to addressing the wicked problem of geographical educational disparity.

## Data Availability

Data for this project are available on request.

## Appendix

Table 7

**Table 7:** Approaches to Primary Science Teaching

Approach	Description
Analogies (Content Representations)	Analogies (Glynn, 2007; Guerra-Ramos, 2011) and Content Represents (CoRes) (Loughran et al., 2004) are often verbal ways of developing primary students’ understanding of an unfamiliar science concept by its similarities with more familiar concepts. Teachers must take care to draw distinctions between where analogies and CoRes are accurate and inaccurate to avoid either creating or reinforcing alternative scientific conceptions (Skamp & Preston., 2021)
Big Ideas/ Inquiry Questions	The ‘Big Ideas’ in science education (Harlen, 2015) inform global science curricula and can be used to frame learning for students. Such big ideas can also be expressed in the form of inquiry questions to create cohesive student learning across activities, lessons, units and year levels. Some science curricula are now framed around inquiry questions (e.g. NESA, 2017); a useful trend given that preservice primary teachers have difficulty forming researchable questions, resulting in superficial experimental designs (Morrison, 2008). Teachers often struggle to afford student choice in the development or selection of science inquiry questions (Biggers, 2018)
Claim-Evidence-Reasoning (CER) Cues	CER cues, either written or verbal, can be used to scaffold primary students understanding of the presentation of claims based on evidence and the associated reasoning needed to connect these two element (Allen & Park Rogers, 2015)
Class Discussions	Class discussions are an essential part of almost all teachers’ teaching repertoires and is often explicitly mentioned in primary science education research (Barnett & Morran, 2002; Liou et al., 2017; Metz, 2008). While class discussions can vary in terms of communicators, communication length, spontaneity and degree of teacher control, they would typically require more than one exchange of information amongst 3 or more participants
Community Projects	Community projects are a means of extending science beyond the typical classroom or school environment. Unlike excursions, community projects often include multiple visits or engagements in service of a broad objective (Keil et al., 2009; Mueller & Bentley, 2009). Community projects can also incorporate multiple visits to the school by community members (Stevens et al., 2016)
Constructivism	Learning that occurs when an individual constructs their knowledge through active cognitive (Kamii & Ewing, 1996) and/or social participation (Vygotsky, 1977) within a phenomenon or situation (Slavin, 1996). Although previously deemed a guiding principle of primary science education (Deehan, 2022), constructivism has been operationalised for teaching through a variety of models and frameworks (Aubusson et al., 2015)
Cross Curricular Integration	An approach to teaching where two discreet disciplines are integrated to create deep learning outcomes. For example, allowing students to collect and graph data is an example of a deep integrative link between mathematics and science (e.g. Kim & Bolger, 2017). Cross curricular integration with science can also occur with art, literacy, music, and drama (Bulunuz, 2013)
Debate	Debate can be seen as a means of advancing more typical science classroom dialogue to a more structured processed base on scientific processes and knowledge (Diakidoy & Kendeou, 2001; France, 2021). They are a rigorous and systematic manner of inducting students into science discourse and the analysis of competing claims (Russell & McGuigan, 2018). Structured debates in primary science classes can positively influence learners’ motivation and science attitudes (Kim, 2019)
Deep Reflection	Deep reflection involves providing support and time for primary learners to consolidate their new, refined science understandings into their existing schemas. This approach is an overarching, intersectional strategy that would typically occur in conjunction with other teaching approaches and would take a variety of forms (brainstorms, reports, group discussions & multimodal representations) (Genc, 2015; Karaçalli and Korur, 2014)
Diagnostic Assessment for Alternative Conceptions	Learners’ alternative conceptions can inform the design and delivery of science learning experiences (McKinnon et al., 2017) and are typically identified through diagnostic assessment at the commencement of a learning and teaching cycle (Celikten et al., 2012; Tarhan et al., 2013). Alternative conceptions can be sourced directly from learners or through scholarly material
Digital Technology/ Simulations	Any form of digital educational technology delivered to primary students with the aim of enhancing science learning. Technological innovations may include; Robots (Shiomi et al., 2015), Technology Enhanced Curriculum (Varma & Linn, 2012), Augmented Reality (Fleck & Simon, 2013), 3d Games (Lester et al., 2014) and Learning Management Systems (Field, 2009)
Direct Instruction/ Transmission	Direct Instruction/ Transmission is the more traditional approach of teacher dissemination of information that places students in a passive recipient role (Jonassen, 1991). It should be noted that direct transmission is often viewed as a necessary part of a balance teaching approach rather than a contradiction of constructivist principles (Godino et al., 2016)
Excursions	Excursions are singular education visits to sites relevant to science education that could not be accessed in a regular school environment. It should be noted that these singular visits can vary in length and can include: excursions to local ecosystems (Prokop et al., 2007), museum visits (Martin et al., 2016), university excursions (Ozogul et al., 2019) and summer camps (White et al., 2018)
Group Work/ Cooperative Learning	Cooperative learning occurs when students work together to complete a task that would otherwise be impossible or unreasonable to complete individually (e.g. Deehan et al., 2017). This strategy is often supported with clear expectations in terms of process and output. For example, students or groups of students may be assigned discreet roles within a larger learning task (Tarhan et al., 2013)
Guest Speakers	Guest speakers are individuals or groups of individuals who are invited into the science learning of primary students, either digitally or physically, to share relevant perspectives, experiences or expertise. Guest speakers are valued complements to regular science teaching practice by students and teachers alike (Flick, 1990; Knobloch et al., 2007)
Hands On Tasks	Hands On Tasks occur when learners are physically engaged in the learning process. Such physical tasks may typically be complemented by an array of consolidative activities (Skamp & Preston, 2021), such as classroom dialogue (Varelas et al., 2006), to ensure science learning objectives are met. Naïve notions of hands on learning can result in activities do not meaningfully advance scientific knowledge or skills (Kleickmann et al., 2016)
Individual Reading	Reading practices are a key component to learning in science and most disciplines. Individual reading has been separated from cross curricular integration for its ubiquity and the rich vein of literacy support research in primary science education, such as varied science texts (e.g. Balim et al., 2016; McTigue, 2009), Concept Oriented Reading Instruction (CORI) (e.g. Guthrie et al., 2004; Wigfield et al., 2008), science language transitions (e.g. Brown & Ryoo, 2008; Brown et al., 2010) and problem solving scaffolds (e.g. Bulu & Pedersen, 2010)
Inquiry Learning	Inquiry learning is characterised by a focus on a specific outcome. It allows participants to apply skills and knowledge to seek the information needed to achieve the outcome. Learners can be afforded partial or complete control of the inquiry process (e.g. Fitzgerald et al., 2019). Inquiry Learning is a means of embodying scientific practice in science learning in alignment with constructivist principles because it requires active, persistent skill use based on personal knowledge (Suduc et al., 2015). Questioning, exploration, making and testing for the acquisition of new knowledge are essential (Lemlech, 2009)
Joint Construction	Joint Construction is the process by which a teacher (expert) works collaboratively to construct a text or product with one or more students (non-experts) (Hermansson et al., 2019); an approach well established in education broadly (Rose and Martin, 2013). It is often a key practice in backward faded scaffolding to gradually increase student independence (Slater et al., 2008). Joint construction has also been linked to positive outcomes in primary science research (Accurso et al., 2016; De Oliveira & Lan, 2014)
Lectures	Lectures are longer periods of direct transmission of teacher knowledge to students in a more passive role. Lectures are commonly associated with more objectivist approaches to teaching (Yarusso, 1992). Despite the long-term shifts to more constructivist modes in science education (Davis et al., 1993), more objectivist approaches such as lectures have still been commonly reported in primary science education (Goodrum, Hackling & Rennie, 2001)
Modelling	This approach is the physical or digital construction of models to reflect scientific knowledge as a means of linking observations, formal or informal, to scientific theory (Schwarz et al., 2009); and has shown to positively influence primary science and general science learning outcomes (Diakidoy et al., 2001; Van Joolingen et al., 2015; Louca & Zacharia, 2012; Shanahan, 2010). Like many other approaches in this framework, modelling intersects with other approaches, such as inquiry (Nersessian, 1995; Windschitl et al., 2008)
Nature of Science Teaching	The understanding that scientific knowledge is fluid and always subject to reasonable debate. Instruction in this area may orient the learner to the variety of scientific approaches beyond an experimental research design (Wilcox & Lake, 2018). Essentially, ‘Nature of Science’ Instruction orients learners to science epistemology (Demirdöğen et al., 2016). Explicit Nature of Science Teaching can enhance the science learning of students engaged in scientific investigations (Lederman & Lederman, 2014), literacy tasks and hands on tasks (Girod & Twyman, 2009)
Note Taking	Note taking is where students record science information, either physically or digitally, for later reinforcement, recall and/or reflection. Note taking can involve rote learning, open student response and/or specific strategies (Lee et al., 2008). Despite being a more passive, objectivist teaching approach, note taking can still be an effective science teaching strategy if effectively supported and incorporated within more complex science lessons or units (Lee et al., 2008). Note taking strategies have also shown to work effectively with classroom tablet technologies (Paek & Fulton, 2016)
Open/ Guided Discovery	Discovery learning is a constructivist approach, well established in science education literature (Balım, 2009; Koksal & Berberoglu, 2014), whereby students come to know the unknown by actively working to construct knowledge based on new data and information made available to them in their learning environment (De Jong & Van Joolingen, 1998; Matson, 2006). The degree of support offered to students engaged in discovery can vary considerably based on context, ranging from guided discovery with consistent guidance, to open discovery, with minimal to emergent guidance (Abd-El-Khalick et al., 2004)
Open/ Higher Order Questioning	Open/ High Order Questions have more than one acceptable answer, with the quality of the answer relating more to a student’s process or reasoning rather than a concrete notion of correctness. Such questions are seen as a mechanism for productive discourse in the science classroom (Chin, 2006, 2007), often in service of science literacy (Roberts & Bybee, 2014), and can be improved through teacher professional development (Caulfield-Sloan & Ruzicka, 2005). Bloom’s Taxonomy (Forehand, 2010; Krathwohl, 2002), Productive questions (Elstgeest, 2001) and the Solo Taxonomy (Biggs & Collis, 2014) can all serve as frameworks for the development of open/ higher order questions. It should also be noted that open and higher order questioning and thinking is closely associated with inquiry and discovery learning (Matthews, 2002)
Other	Other was included in the framework to acknowledge the impossibility of capturing all possible science teaching approaches by allowing teachers to present approaches of which the research team may not be aware
Outdoor Science	Outdoor science occurs when students are able to authentically engage with environments beyond the classroom for the purpose of meaningful science learning (Assaraf & Orion, 2010). Supplementing classroom science with outdoor learning experiences can significantly improve primary students’ science knowledge (Prokop et al., 2007) and skills (Ting & Siew, 2014). However, primary teachers have reportedly viewed outdoor science experiences to difficult and inefficient to implement due to time constraints and demanding curricula (Carrier et al., 2013)
Peer Tutoring	Peer Tutoring involves at least one student assisting (i.e. the tutor) at least one other student to achieve (i.e. the tutee) a relevant science learning objective in a defined period of time (Stephenson & Warwick, 2001). Such tutoring can occur within and across classes and can be adjusted to suit changing learning needs and objectives. Research has shown that peer tutoring can improve the science content knowledge and attitudes of both tutors and tutee (Topping et al., 2004)
Predict-Observe-Explain (POE) Cues	Predict-Observe-Explain (POE) cues are foundational scaffolds to frame students’ sensory data (Observe) in science thinking (Jasdilla et al., 2019). Research has shown that such cues can aid conceptual change in primary science learners (Dial et al., 2009; Westman & Whitworth, 2019). POE cues can also function as catalysts for inquiry learning (Liem, 1990)
Project/ Problem-Based Learning	Project/ Problem-based learning uses real-world problems as a starting point for the acquisition and integration of new knowledge into existing schemas (Etherington, 2011; Keil et al., 2009; Sari et al., 2018). This approach to science education helps students to develop transferable skills which can be used in novel situations. Project/ problem based learning can be characterised by the requirement for systems thinking, that often links science to other learning areas, and a lack of immediate clarity regarding the ‘correctness’ of actions or answers (Stepien & Gallagher, 1993). In essence, such open ended approaches eschew the relative linear, narrative structure of other approaches (Drăghicescua et al., 2014)
Science in the Media	Engaging with Science in the Media is a way for primary students to explore socioscientific issues to better understand how science influences societies (Dolan et al., 2009; Presley et al., 2013). Such approaches enable primary learners to engage with important issues, such as: pest eradication, water security and climate change, with supplementary approaches such as debate, group learning, digital technologies (Evagorou et al., 2015; Grumbach, 2019; Kahn & Hartman, 2018). The research on science in the media and associated socioscientific issues has increased considerably over the past decade (Tekin et al., 2016)
Second Hand Research	Second hand research involves students analysing data and/or interpreting information that they themselves did not collect directly. Such second hand research can effectively complement first research experiences to show students how science relates to the world beyond the classroom and improve their scientific knowledge and reasoning (Palincsar & Magnusson, 2001). While, subsequent classroom research has shown that second hand research unique benefits for the development of scientific inquiry skills (Hug & McNeill, 2008), it cannot replace first hand research experiences (Delen & Krajcik, 2015)
Station Rotation	Station rotation involves the set up and delivery of separate, but possibly related, learning activities (stations) that students can worth through, individually or cooperatively, for periods of time during a science learning experience. These series of activities can be timed or self-paced (Martin et al., 2016). Research has shown that station rotations can covary with highly significant improvement to primary students’ science achievement and skill (Ocak, 2010)
Student Centred Investigations	Student Centred Investigations are one of the broader conceptualisations of scientific investigation in education research. For an investigation to be considered student centred, full or partial student input into the purpose, parameters, process and/or consolidation of a scientific investigation must be offered. Student centred investigations intersect with other approaches and vary considerably in term of form and function within the science education research literature (Aydede & Matyar, 2009; Quigley et al., 2010; Skamp & Preston, 2021)
Teacher Demonstration	Teacher demonstration occurs when a teacher represents the science related actions, skills, knowledge and/or dispositions they wish their students to emulate or embody. Although teacher demonstration is a longstanding approach (Glasson, 1989; Goodrum et al., 2001) often considered to be a more passive teaching approach, it has been linked to improve science learning outcomes (Shepardson et al., 1994) and can be foundational element to more complex science programs (Ozogul et al., 2019)
Teacher Lead Investigations	Teacher lead investigations contrast directly with student centred investigations as they offer students with no meaningful input into any phase of a scientific investigation. Often referred to derisively as “on rails” or “cook book” investigations (Özgelen et al., 2008; Şeşen & Mutlu, 2016), teacher lead investigations are often cast as the “traditional approaches” used for control groups in science education research (Balim et al., 2016; Durmuş and Bayraktar, 2010; Girod et al., 2010). However, teacher lead investigations can be seen as efficient solutions to resource and time limitations in primary school and beyond (Deehan, 2022; Carrier et al., 2013)
The 5Es Framework	The 5Es framework underpins the Primary Connections resources (e.g. AAS, 2011, 2012a, b, 2019) that are frequently used in Australian primary schools (Albion & Spence, 2013; Aubusson et al., 2019; Hume, 2012) and are well-supported by academic research. This commonly used framework is comprised of five phases: Engage, Explore, Explain, Elaborate and Evaluate (AAS, 2019; Bybee et al., 2006; Bybee, 2015)
Watching Videos	Teachers can often provide videos to provide students with information and insights that they would not otherwise be able to access in a classroom environment. There is considerable variation in how videos can be contextualised within science learning sequences; ranging from time fillers to thoughtful catalysts and/or consolidators of active science learning. For example, Chen and Cowie (2014) showed that videos of scientists can engage a wide array of science learners. Recent research has also show that motion graphic animation videos can help to improve primary students’ science achievement (Hapsari & Hanif, 2019). Further to this point, Koto (2020) found that the inclusion of thoughtfully curated YouTube videos into a discovery learning program can significantly improve Year 5 students’ factual, procedural and conceptual knowledge of heat transfer. It is also important to note that videos can be an efficient way of addressing both longstanding (resourcing & time) and emergent (e.g. Covid-19) barriers
Worksheets	A mainstay of science education (Goodrum et al., 2001); physical or digital worksheets provide written and visual cues for students to respond to in a science learning context. Despite being considered a passive learning approach, there is some evidence that worksheets can be effective in consolidating learning (Johnson et al., 1997). Worksheets have been found to be beneficial in informal science education settings (Mortensen & Smart, 2007; Nyamupangedengu et al., 2012) and augmented reality learning environments (Zhang et al., 2020). Worksheets continue to feature prominently in primary science education interventions (Deehan et al., 2022)
